# Properties investigations of rape stalks fermented by different salt concentration: Effect of volatile compounds and physicochemical indexes

**DOI:** 10.1016/j.fochx.2023.100746

**Published:** 2023-06-10

**Authors:** Sijie Zhang, Congcong Li, Junling Wu, Simin Peng, Weiguo Wu, Luyan Liao

**Affiliations:** College of Food Science and Technology, Hunan Agricultural University, Changsha 410128, China

**Keywords:** Fermented vegetables, Natural fermentation, Antioxidant components, Free amino acids, Characteristic volatile components, Cluster analysis

## Abstract

In order to find out the effect of salt concentration on fermented rape stalks, the physicochemical quality and volatile components was investigated using high performance liquid chromatography (HPLC) and headspace solid phase microextraction gas chromatography-mass spectrometry (HS-SPME-GC–MS). The results showed that there were abundant kinds of free amino acids (FAAs) in all samples, mainly presenting sweet, umami and bitter taste. Through taste activity value (TAV), His, Glu, and Ala contributed significantly to the taste of the sample. 51 volatile components were identified, of which the relative contents of ketones and alcohols were high. By the relative odor activity value (ROAV) analysis, the main components that had a great impact on the flavor were phenylacetaldehyde, β-Ionone, ethyl palmitate and furanone. Adjusting the appropriate salt concentration for fermentation could improve the comprehensive quality of fermented rape stalks and promote the development and utilization of rape products.

## Highlights

• The physicochemical quality changed greatly before and after fermentation.
• The free amino acids in fermented rape stalks were very rich.
• The detected various components formed the special flavor of fermented rape stalks.
• The use of different salt concentration fermentation had a significant impact on the comprehensive quality of rape stalks.

## Introduction

Rape is the most widely distributed and sown oil crop in China. Rapeseed produced by rape is the raw material of rapeseed oil production. Rape stalks were vegetables, fresh and tender, rich in vitamin C, carotene, protein, etc. However, due to the high water content of fresh rape stalks and short shelf life, rape can be made into fermented vegetables. At present, the famous fermented vegetables mainly include Korean kimchi (Jung, Lee, & Jeon, 2014), Chinese northeast pickle (Zhou, Zang, Zhao, & Li, 2018), American pickled cucumber (Pérez-Díaz, Hayes, Medina, Webber, Butz, Dickey, et al., 2019), etc.

Fermented vegetables have better flavor, more outstanding quality in taste, storage time and nutrition. At the same time, it is convenient for product packaging and transportation. It can better adjust the shortage period of vegetables. Fermented vegetables which good for human health, having great effect on maintaining intestinal health, anti-inflammation and sterilization, anti-arteriosclerosis, anti-obesity, lowering cholesterol and improving immunity (Irakoze, Wafula, & Owaga, 2021). The salt was always used to preserve the fermented vegetables. On the one hand, salt has antiseptic effect and can inhibit the activities of harmful microorganisms and enzymes. On the other hand, the penetration of salt can dehydrate vegetable tissues and exude soluble nutrients such as sugars, amino acids, and vitamins (Yang, Niu, Shan, Zheng, Liu, Wang, et al., 2021). An appropriate amount of salt not only increased the saltiness and freshness of the product, and affects the oxidation and decomposition of nutrients such as protein and lipid (Xu, Jin, Su, Yang, Xu, Jin, et al., 2021). Therefore, there is a significant impact on the quality and flavor of the product when adding the salt. But intaking too much salt increased the risk of cardiovascular and cerebrovascular diseases (Narula, McKee, Martin, Mancia, & Giuseppe, 2017) The selection and control of salt concentration range was an important factor affecting the product quality and safety in the process of vegetable fermentation (Bonatsou, Iliopoulos, Mallouchos, Gogou, Oikonomopoulou, Krokida, et al., 2017).

Different concentrations of salt had different effects on the reproduction and metabolism of lactic acid bacteria in fermented vegetables, affecting their maturity and the utilization of sucrose, resulting in different physical and chemical indexes and different effects on the sensory and flavor of products. The content of nitrite and volatile ester in fermented vegetables decreased with the increase of salt content, and the pH increased, while FAAs decreased first and then increased. Under the action of microbial hydrolase and protease, the protein in vegetables was decomposed into various amino acids, which made the fermented vegetables taste more delicious. At present, there is no research on the technology of using rape stalks as the raw material of vegetable fermentation. Therefore, the study about the effect of salt concentration on the quality of fermented rape stalks is of great significance to the development of rape industry.

In this paper, rape stalks were taken as the research object. The effects of different salt concentrations on the physicochemical properties and flavor characteristics of wet cured rape stalks were compared, and fresh rape stalks was used as a control, which provided a theoretical reference for the selection of salt concentration in the process of rape stalks pickling and improving the product quality. The aim of the study was laying a foundation for the follow-up study of rape stalks series products and the improvement of rape stalks fermentation process.

## Materials and methods

### Preparation and collection of fermented rape stalks

Fresh rape stalks (*Brassica napus*) were collected from Crop Center in Changsha, Hunan Province. Rape stalks was washed and cut into 3 cm lengths. The moisture content of fresh rape stalks was 60% ± 2% in a hot air drying oven (50 °C). The rape stalks were put into a sterilized ceramic pot, and salt water of different concentrations (the weight ratio of raw material to brine was 2:3) was added to it, and they were stirred evenly. The concentrations of brine were 12%, 14%, 16%, 18% and 20%, respectively, and three parallel groups were made for each concentration. Then the plastic wrap was covered on the rape stalks. The sterilized smooth stones were pressed on the rape stalks to ensure the anaerobic environment for the growth of lactic acid bacteria. The jar mouth was sealed with fresh-keeping film, the bottle cap was closed, and then water was added for fermentation. The jar was put into a constant temperature(20 °C) environment for fermentation(30 days). The finished product of fermented rape stalks were freeze-dried by vacuum freeze dryer, crushed into powder, screened by 80 mesh, vacuum packaged, and stored in − 80 °C refrigerator for standby.

### Determination of physicochemical properties

The content of soluble sugar was determined by anthrone colorimetry. The results were calculated based on the standard curve obtained from a 0.00 to 0.15 mg/mL glucose solution. Fat content was determined by Soxhlet extraction method (Luque-García & Luque de Castro, 2004). Petroleum ether was used as the extraction solvent. Determination of protein by Kjeldahl method. The automatic Kjeldahl nitrogen analyzer (Shandong Haineng Scientific Instrument Co., Ltd., Jinan, China) was used to determine the crude protein content. Amino acid nitrogen was determined according to the formaldehyde value method (Ouyang, Chen, Zhao, & Lin, 2013).

Total phenols were determined with reference to Folin phenol colorimetry (Xu, Du, & Xu, 2015), which was slightly modified. A 5 g sample was taken and ground with 75% ethanol and then ultrasonically extracted for 1 h. Total phenols content values of different samples were calculated based on a calibration curve of gallic acid. The content of flavonoids was determined with reference to the aluminum trichloride colorimetry (Rahman, Abdullah, Sharif, Jahan, Kabir, Motalab, et al., 2022), and a little modification was made. Flavonoids in the sample were extracted using 70% ethanol with ultrasonication for 30 min. A calibration curve was plotted with a rutin standard dilution series.

### Determination of FAAs

FAAs of fermented rape stalks were determined by HPLC (Agilent 1100, Agilent, CA, USA) with some modifications(Yao, Ma, Wu, Wang, Xu, Yu, et al., 2022). Using a ZORBAX Eclipse AAA column (4.6 × 75 mm, 3.5 μm, Agilent, CA, USA). UV detection was equipped with 338 nm (0–19 mins) and 266 nm (19.01–25 min). Mobile phase A, 40 mM sodium dihydrogen phosphate (pH 7.8); mobile phase B, acetonitrile/methanol/water = 45/45/10; An eluant flow rate of 1.0 mL/min. TAV was calculated by the ratio of the content of each FAA to its taste threshold.

### Analysis of volatile components

The volatile compounds in the rape stalks were analyzed using HS-SPME/GC–MS spectrometry (QP 2010 PLUS, Shimadzu, Japan). 1.0 g sample was placed in 20 mL headspace bottle and extracted with fiber head (65 µ m Polydimethylsiloxane / Divinylbenzene) (PDMS/DVB, Supelco, Inc., Bellefonte, PA, USA) for 30 min at 70 °C. Then the extraction head is inserted into GC–MS for 5 min. Chromatographic column (Rtx-5MS, 30.0 m × 0.25 mm × 0.25 µ m, Shimadzu Co., Kyoto, Japan) for analysis of volatile components. Heating program: the initial column box temperature was 40 °C, which was maintained for 3 min, then rose to 150 °C at 3 °C /min, which is maintained for 4 min, and then rose to 230 °C at 10 °C /min, which was maintained for 5 min. Mass spectrometry conditions: ion source temperature 200 °C, detector voltage 1.2 kV, interface temperature 220 °C, scanning range 50–500 *m*/*z*, solvent delay time 5 min.

Qualitative analysis of volatile components was based on comparisons of the National of standards and Technology (NIST17) mass spectral libraries and by matching the retention index (RI) values. When the matching degree was greater than 80%, the identification result would be used (up to 100%). The peak area normalization method was used to quantitatively calculate the relative concentration of volatile components. The ROAV was calculated using the following formula, which can evaluate the volatile components:

$${\text{ROAV}}_{\text{a}}=100\times {\text{OAV}}_{\text{a}}/{\text{OAV}}_{\text{max}}$$

where ROAV_a_ is the relative odor activity value of volatile component_a_; OAV_max_ is the highest odor activity value (OAV) among the volatile components; and OAV_a_ is the OAV of volatile components_a_. OAV was calculated via the equation OAV_a_ = C_a_/T_a_, where C_a_ was the relative concentration and T_a_ was the odor threshold.

### Statistical analysis

Analyses of basic physicochemical index results were performed in triplicate. Origin 2018 and SPASS 25 software were applied for analysis of significance and statistical results. The significant differences among means were analyzed using an analysis of One-Way (ANOVA). Duncan test was utilized to determine the significant differences among samples(the significance level p＜0.05).

## Results and discussion

### Changes of physicochemical properties

The soluble sugar, fat, protein and amino acid nitrogen of fresh rape stalks during fermentation with different salt concentrations are shown in Table 1. The soluble sugar content of the 12% salt samples was the lowest (6.47 ± 0.29 mg/100 g), and the soluble sugar content of the fresh sample was as high as 53.33 ± 0.77 mg/100 g. After fermentation, it was significantly lower than the fresh samples. It is vital for microbial to use carbohydrates for growth and metabolism (de la Fuente, Luz, Puchol, Meca, & Barba, 2021). Some microorganisms decomposed fiber, starch and other substances to increase soluble sugars (Quan, Liu, Guo, Ye, & Zhang, 2022). The content of soluble sugar in the fresh samples was higher than that in fermented samples, the result was similar to the study of (Tian, Deng, Zhao, Du, Li, Lai, et al., 2021). The fat content of the 12% salt samples was the highest (4.55 ± 0.13 g/100 g). Generally, the fat content decreased with the increase of salt concentration for fermentation. There were significant differences in the fat content measured after fermentation under the five salt concentrations. During fermentation, lactic acid bacteria decomposed fat under the action of extracellular enzymes to produce aldehydes, ketones and other substances related to fermentation flavor.

**Table 1:** Basic physicochemical indexes of fresh and fermented rape stalks.

Sample	Soluble sugar(mg/100 g)	Fat(g/100 g)	Protein(g/100 g)	Amino acid nitrogen(g/100 g)
Fresh samples	53.33 ± 0.77^a^	3.75 ± 0.13^b^	30.62 ± 0.08^a^	2.14 ± 0.13^a^
12%	6.47 ± 0.29^f^	4.55 ± 0.13^a^	16.46 ± 0.14^d^	1.32 ± 0.01^b^
14%	16.91 ± 0.84^c^	3.80 ± 0.02^b^	17.50 ± 0.33^c^	1.15 ± 0.10^c^
16%	10.54 ± 0.26^e^	2.29 ± 0.03^d^	22.44 ± 0.10^b^	1.16 ± 0.09^c^
18%	13.54 ± 0.21^d^	3.23 ± 0.04^c^	13.47 ± 0.31^e^	0.83 ± 0.03^d^
20%	18.40 ± 0.61^b^	1.62 ± 0.13^e^	13.25 ± 0.01^e^	0.59 ± 0.04^e^

Different letters (a, b, c, e and f) in the same line indicate significant differences (P＜0.05).

The protein content of the fresh samples was as high as 30.62 ± 0.08 g/100 g, which was rich. The protein content of each sample after fermentation was lower than the fresh samples. With the increase of fermented salt concentration, the protein content first increased and then decreased, and there was a peak in the 16% salt samples. Some studies have proved that fermentation can significantly improve the digestibility of protein, and the digestible protein had better nutritional value than the non-digestible protein (Alrosan, Tan, Mat Easa, Gammoh, & Alu’datt, 2021). Some lactic acid bacteria had a protein hydrolysis system that induced protein degradation to peptides (Rui, Huang, Xing, Zhang, Li, & Dong, 2019). Some strains had high protein hydrolysis ability, and protein hydrolysis affected the utilization of amino acids. After fermentation, the content of water-soluble proteins and amino acids increased, which made it have a fresh taste, and nutrients were more easily absorbed by the human body (Su, Jiang, Hao, Li, Gong, Zhang, et al., 2021). The content of amino acid nitrogen was the key parameter of rape stalks fermentation. The content of amino acid nitrogen reflected the degree of protein hydrolysis, made the fermented rape stalks taste soft and reconciled the aroma. The lower the concentration of salt used in fermentation, the higher the content of amino acid nitrogen, which might be related to protease activity. At higher salt concentration, the high osmotic pressure of fermentation environment would inhibit the metabolic activities of lactic acid bacteria and other microorganisms, while low salt fermentation could accelerate the maturity of products (Xiong, Li, Liang, Wang, Guan, & Xie, 2016). The process of protein degradation was accompanied by more complex biochemical reactions between this small molecule amino acid and other substances, resulting in the reduction of amino acid nitrogen (Chen, Zeng, Liao, Li, Zhou, Yang, et al., 2020).

### Analysis of changes of antioxidant active components

**Fig. 1:**
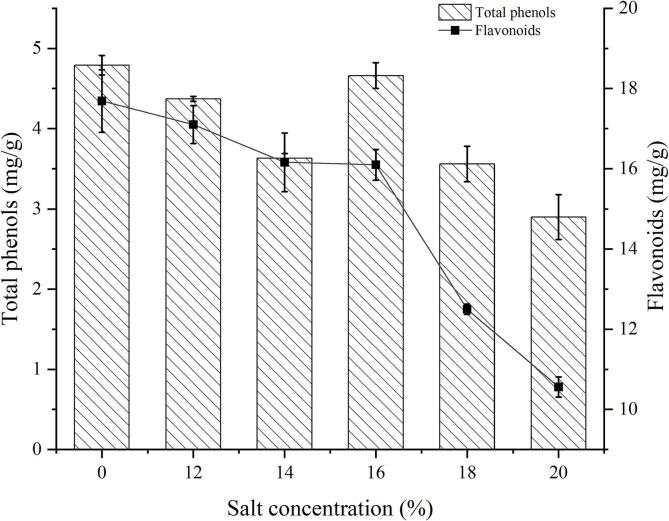
Content of total phenols and flavonoids in fresh and fermented rape stalks under different salt concentration (12,14,16,18 and 20 represented 12%, 14%, 16%,18% and 20% salt addition (w/w), 0 represents the fresh samples.).

Phenolic compounds were the most effective antioxidants in plants at present. Total phenols and flavonoids were considered to be the main antioxidant active components (Aryal, Baniya, Danekhu, Kunwar, Gurung, & Koirala, 2019), which benefited from the phenolic hydroxyl structure. The hydrogen donor scavenged a variety of reactive oxygen species and generate polyphenol free radicals with low activity to achieve the effect of antioxidation. The contents of total phenols and flavonoids in fresh and fermented rape stalks with different salt concentrations were shown in Fig. 1. The total phenol content in the fresh samples was the highest, reaching 4.79 mg/g. After fermentation, it showed an overall downward trend with the increase of concentration, but there was a peak (4.66 mg/g) close to the fresh samples at the 16% salt samples. The flavone content of the fresh samples was up to 17.69 mg/g, which decreased with the increase of salt concentration after fermentation. The significant decline period of flavone content was mainly concentrated in the 16%, 18% and 20% salt samples. Different materials, manufacturing process and fermentation environment might cause the above marked differences in antioxidant active components contents.

Rape stalks contained a high content of protein, but some studies have shown that phenolic compounds and proteins had covalent interaction, resulting in the further reduction of total phenolic content and antioxidant properties (Wang, Li, Zhu, Liu, Liu, Yu, et al., 2021). The appropriate concentration of salt allows specific microorganisms to secrete more enzymes such as proteases and amylases, which were beneficial for promoting the release and formation of antioxidant compounds such as total phenols, flavonoids, and FAAs during fermentation, thereby enhancing antioxidant activity (Chen, Feng, Wang, He, Xu, Gao, et al., 2022). There was a small amount of oxygen in the fermentation container, phenols were oxidized to form quinones, and the total phenol content would also be reduced under the action of polyphenol oxidase (Kasnak, 2020). Some researchers found that polyphenol oxidase activity decreased during fermentation (Liczbiński, & Bukowska, 2022), and incomplete fermented white tea and green tea contained more polyphenols, which was similar to our conclusion. Sodium ion in salt affected the activity of polyphenol oxidase. The increase of lactic acid bacteria and osmotic pressure during fermentation affected the oxidation, reduction or degradation of phenolic compounds. Microbial post fermentation reduced the content of flavonoids or convert them into other substances (Li, Wang, Ma, Yang, Chen, Yu, et al., 2021).

### Analysis of FAA changes

**Fig. 2:**
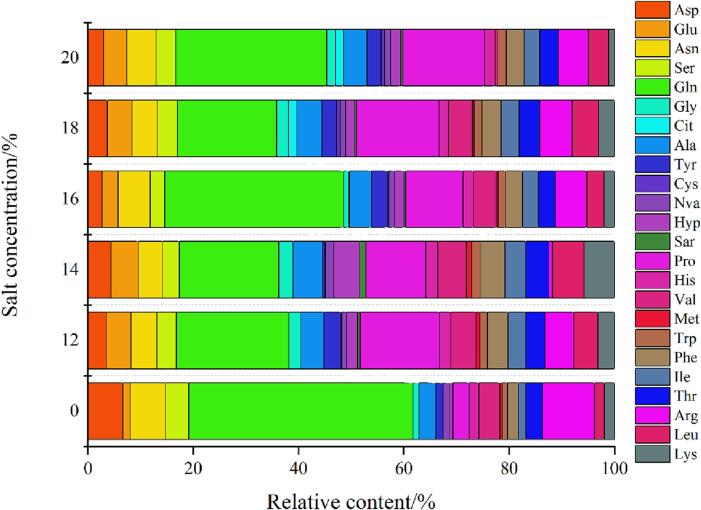
Composition and content of FAAs in fresh and fermented rape stalks with different salt concentration (12,14,16,18 and 20 represented 12%, 14%, 16%,18% and 20% salt addition (w/w), 0 represents the fresh samples.).

FAAs was a kind of important bioactive substance which exists in free state in cell and had important physiological function and medicinal value (Guo, Duan, Qian, Tang, Qian, Wu, et al., 2013). Table S2 showed the detected FAAs, A total of 6 essential amino acids (His, Val, Met, Trp, Ile), 14 non-essential amino acids (Asp, Glu, Asn, Ser, Gln, Gly, Cit, Ala, Tyr, Cys, Nva, Hyp, Sar, Pro) and 4 semi-essential amino acids (Thr, Arg, Leu, Lys) were detected. Fig. 2 helped to better understand the content distribution of FAAs in each sample. Gln (mean 6.818 mg/g) and Pro (mean 3.821 mg/g) were the highest in the six samples. Gln was glutamic acid amide, slightly sweet, was used for the treatment of gastritis, and improved the brain function of children with poor intellectual development, as the most abundant FAAs in human muscle, could regulate blood sugar and nutrition metabolism (Cruzat, Rogero, Keane, Curi, & Newsholme, 2018). The increase of Pro in rape stalks might be related to the concentration of sodium ion and chloride ion under saline conditions (Forlani, Bertazzini, & Cagnano, 2019). Pro was mainly used as a clinical treatment for malnutrition, protein deficiency and severe gastrointestinal diseases. Cys content (mean 0.092 mg/g) was the lowest and did not exist in the fresh samples. After fermentation, new physiological active substances such as certain amino acids could be produced, and taste could be significantly improved (Zhu, Luo, Wang, Zhao, Li, Hu, et al., 2016). Cit was not found in the fresh samples and low salt fermentation samples, and Met was not found in the 20% salt samples. Low salt fermentation increased the content of essential amino acids, up to 6.637 mg/g, making it more nutritious. With the increase of fermentation salt concentration, the content of essential amino acids decreased, and the content of total FAAs also decreased. Some studies found that the number of FAAs decreased after the interaction between phenolic compounds and proteins (Omar, Arken, Wali, Gao, Aisa, & Yili, 2022). FAAs were used as an index to measure the degree of protein degradation. Fermentation led to protein degradation, which improved physicochemical properties and antioxidant activity (Zhang, Zhang, Xie, & Sun, 2021). Due to the hydrolysis of protein by microbial enzymes, proteins were decomposed into polypeptides and a variety of FAAs (Bao, Zhang, Zheng, Ren, & Lu, 2018). The salt concentration in brine not only affected the flavor of fermented products, but also affected their microbial community structure, thus affecting the content of biogenic amines and FAAs (Świder, Wójcicki, Bujak, Juszczuk-Kubiak, Szczepańska, & Roszko, 2021).

**Fig. 3:**
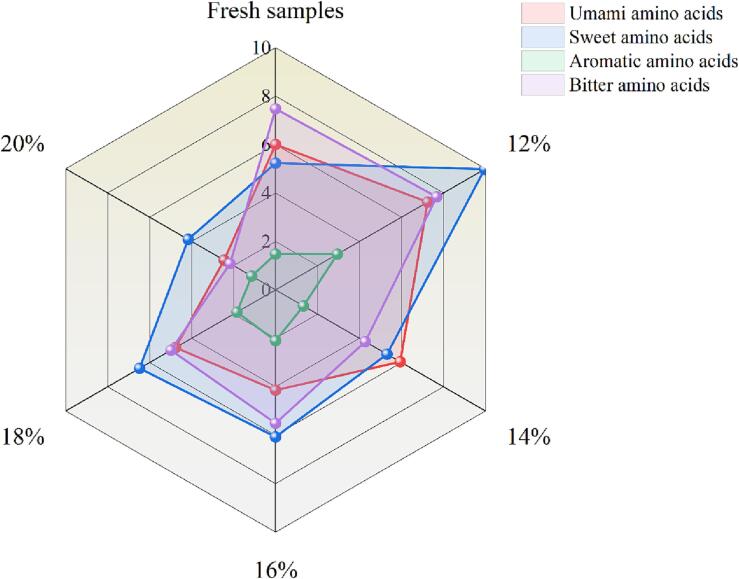
Radar map of flavor amino acids in fresh and fermented rape stalks with different salt concentrations.

FAAs are essential for the flavor profile of the products, which presented various flavors such as umami, sweet, bitter and so on. Since the taste threshold of each amino acid was different, it could not only be judged from the content. The TAV combines threshold and content, the larger the TAV, the greater the contribution of this FAA to taste. When the TAV is greater than 1, it indicates that it has a significant flavor contribution, but the taste sensitivity towards FAAs of subjects from different countries or districts is usually quite different (Gao, Zhang, Liu, Yang, Chen, Hu, et al., 2019). The TAV of fresh and fermented rape stalks under different salt concentrations were shown in Table S3. The TAV of Glu and Ala in the umami amino acids were greater than 1 in each sample, only His in the sweet amino acids was greater than 1, mostly taste thresholds were relatively low. Fermentation improved the contribution of Pro and Glu to the taste of rape stalks, but decreased the TAV of Arg and Asp. Combined with the radar chart in Fig. 3, sweet amino acids were prominent in most samples after fermentation. The bitter amino acids in fresh samples were more prominent. The bitter amino acids might come from uncoordinated protein hydrolysis (Yang, Xia, Wang, Tao, Yu, & Ai, 2019). But the umami amino acids fermented at 14% salt concentration were more obvious, and the TAV of Asp, Glu, Ala and Lys were greater than 1. The content of bitter and umami amino acids in the 12% salt samples were the highest. The proportion and content of aromatic amino acids were small. The content of bitter amino acids was also higher in rape stalks with high content of fresh and sweet amino acids. It was seen that the proportion of various flavor amino acids of rape stalks fermented with different salt concentration was different. They cooperated and worked together to give the fermented rape stalks unique taste characteristics.

### Analysis of volatile components

The sensory threshold, ROAV and odor description of 51 representative volatile components were shown in Table S4. The volatile components of rape stalks fermented under different salt concentrations were determined by HS-SPME and coupled GC–MS, including alcohols, aldehyde, acids, hydrocarbon, ketones, esters and so on. The contribution of volatile components to rape stalks flavor was compared using ROAV calculated by content and sensory threshold. Most volatile compounds were not perceptible due to their low concentrations and/or high threshold values (Xiao, Wu, Niu, Wu, Zhu, Zhou, et al., 2017). Generally, components with ROAV ≥ 1 were the main flavor components, and components with 0.1 ≤ ROAV < 1 played the role of modification and coordination. The odor description of volatile components could more fully analyze the main flavor of the sample. It was found that the ROAV values of acids, alcohols, hydrocarbon and heterocycles were less than 0.1, and the contribution to the flavor of rape stalks was small. There were 12 volatile components in the fresh sample with ROAV ≥ 1, however, after fermentation with medium salt concentration, only 3 to 4 volatile components with ROAV ≥ 1 were found. The main contributing volatile components in fresh samples were β- Ionone, nonanal, phenylacetaldehyde, 1-octen-3-ol, decanal, pentadecanal, and so on. 1-octene-3-ol mainly provided green fragrance and vegetable fragrance. High ROAV values were detected in dimethyl trisulfide at salt concentrations of 14%, 16%, 18% and 20%, but not in fresh and 12%, which mainly contributed to areca fragrance. Decanal had a fatty and fruity aroma such as citrus. Phenylacetaldehyde also contributed greatly to the flavor of the fermented samples. Ethyl palmitate was only detected after fermentation, providing a creamy and ester flavor. The main flavor components of fermented rape stalks were mainly phenylacetaldehyde, β- Ionone, furanone, methyl palmitate and ethyl palmitate, etc. After fermentation, some new volatile components appeared compared with the fresh samples, which gave the fermented rape stalks a unique flavor.

**Fig. 4:**
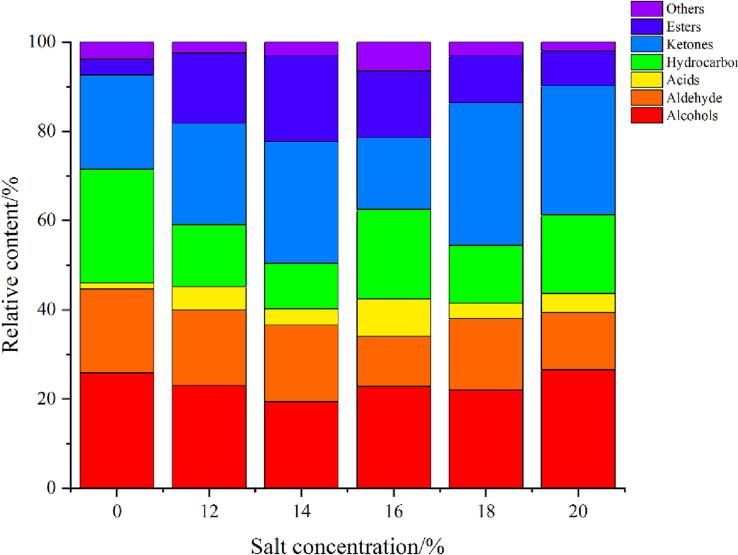
Types and relative content of volatile components of fresh and fermented rape stalks under different salt concentrations(12,14,16,18 and 20 represented 12%, 14%, 16%,18% and 20% salt addition (w/w), 0 represents the fresh samples.).

The types and relative content of volatile components in each sample were shown in Fig. 4. Ketones mostly came from the oxidative degradation of unsaturated fatty acids, the threshold was large, it had little contribution to flavor. Aldehydes were mainly produced by amino acid degradation. Phenylacetaldehyde could be derived from the oxidative degradation of phenylalanine (Wang, Zhang, Kong, Peng, Li, Liu, et al., 2017), giving fermented rape stalks nut fragrance; Esters reach the peak at 14% salt concentration, and then the percentage of esters decreased significantly with the increase of salt concentration. Ethyl laurate, ethyl palmitate and methyl palmitate were common in rapeseed oil and mainly provide cream and fruit aroma; The proportion of hydrocarbons was large, but most hydrocarbons with more carbon atoms could modify the flavor, and their threshold was large, which had little contribution to the flavor of rape stalks. The formation of fermented flavor is a complex process in which microorganisms and the environment work together in the fermentation process. In conclusion, the production and relative content of volatile components were significantly affected by different salt concentrations.

### Cluster analysis of FAAs

**Fig. 5:**
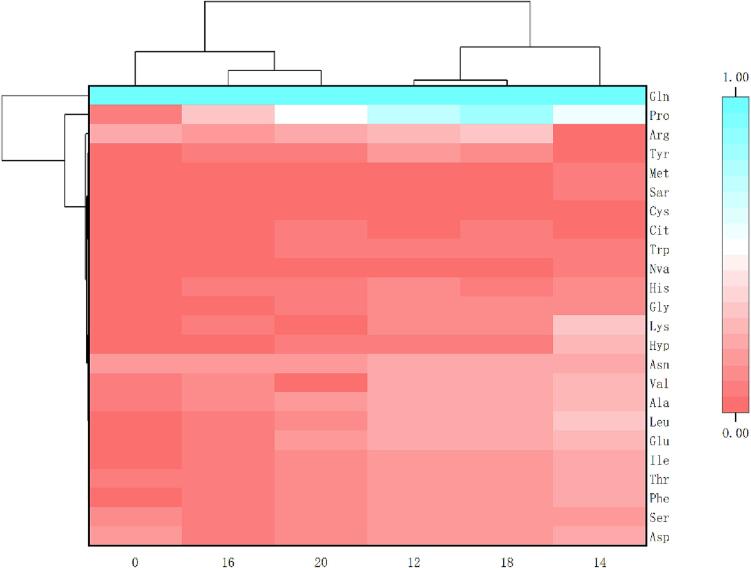
Cluster analysis of fresh and fermented rape stalks under different salt concentrations(12,14,16,18 and 20 represented 12%, 14%, 16%,18% and 20% salt addition (w/w), 0 represents the fresh samples.).

FAAs in fresh and fermented rape stalks were further analyzed, and the square Euclidean distance was used as the measurement criterion for cluster analysis. As shown in Fig. 5, the samples were clustered horizontally, and the types of FAAs were clustered vertically. The original data were standardized. The clustering of the same kind indicated the high degree of correlation between them, and the shorter the Euclidean distance indicated the higher degree of correlation.

From the clustering results, the horizontal cluster was further divided into four subclusters. Subcluster 1 was the fresh samples. Subcluster 2 was composed of samples with 16% and 20% salt concentration. Subcluster 3 was composed of samples with 12% and 18% salt concentration. Subcluster 4 was samples with 14% salt concentration. Samples with 12% and 18% salt concentration were clustered, and it represented that the high correlation between the samples fermented with 12% and 18% salt concentration. According to the clustering results of FAAs, they were summarily divided into three collections. High content of FAAs (Glu, Pro) was expressed by blue, followed by white and red series with the decrease of FAAs content. Glu and Pro are grouped into one class respectively, and other FAAs are grouped into one class. The results showed that the content of FAAs increased after fermentation, and the samples with 14% salt concentration were higher. Except Glu and Pro, other FAAs showed strong correlation.

## Conclusion

In this study, the effects of different salt concentrations on the basic physicochemical indexes, antioxidant active components, FAAs and volatile compounds of naturally fermented rape stalks were investigated in detail for the first time, and compared with the fresh samples. High salt fermentation reduced the content of fat, protein and amino acid nitrogen. Rape stalks fermented under 14% salt concentration had outstanding umami amino acids and higher content of soluble sugar and protein. All samples were rich in FAAs and high in Glu and Pro, which had potential medicinal development value. The main volatile components were aldehydes, ketones, hydrocarbons, esters and alcohols. After fermentation, the number of volatile components increased significantly, endowing fermented rape stalks with a unique flavor. Cluster analysis was performed on FAAs of all samples, and it was found that Glu and Pro were grouped together and distinguished from other FAAs and the sample fermented at 14% salt concentration was also clustered separately. The results may be helpful to develop new fermented vegetables with appropriate salt concentration (using rape stalks as raw material) and fully develop rape resources at bolting stage. Future research should focus on the correlation between the fermentation conditions (such as fermentation time, temperature, etc.) and the overall quality of fermented rape stalks. At the same time, the potential mechanism of the core functional microorganisms affecting the formation of its characteristic flavor still needs to be more comprehensive and in-depth research using more advanced technologies.

## Declaration of Competing Interest

The authors declare that they have no known competing financial interests or personal relationships that could have appeared to influence the work reported in this paper.

## Data Availability

Data will be made available on request.
