# Supplementary material for: Properties investigations of rape stalks fermented by different salt concentration: Effect of volatile compounds and physicochemical indexes
Source: Food Chem X. 2023 Jun 10;18:100746. doi: 10.1016/j.fochx.2023.100746 (PMC10314211; doi:10.1016/j.fochx.2023.100746)
Supplement: Supplementary data 1 [file mmc1.docx]

**Table S2 Composition and content of FAAs in fresh and fermented rape stalks with different salt concentrations**

| Composition | The fresh samples | | 12% | | 14% | | 16% | | 18% | | 20% | |
| --- | --- | --- | --- | --- | --- | --- | --- | --- | --- | --- | --- | --- |
|  | Content（mg/g) | Percentage (%) | Content（mg/g) | Percentage (%) | Content（mg/g) | Percentage (%) | Content（mg/g) | Percentage (%) | Content（mg/g) | Percentage (%) | Content（mg/g) | Percentage (%) |
| Nonessential amino acids | 30.6 | 72.42 | 27.007 | 66.78 | 16.088 | 64.12 | 23.116 | 71.21 | 17.184 | 66.76 | 12.663 | 75.34 |
| Asp | 2.811 | 6.65 | 1.44 | 3.56 | 1.092 | 4.35 | 0.867 | 2.67 | 0.957 | 3.72 | 0.501 | 2.98 |
| Glu | 0.581 | 1.38 | 1.879 | 4.65 | 1.309 | 5.22 | 0.988 | 3.05 | 1.216 | 4.72 | 0.746 | 4.44 |
| Asn | 2.847 | 6.74 | 1.99 | 4.92 | 1.167 | 4.65 | 1.987 | 6.12 | 1.23 | 4.78 | 0.935 | 5.56 |
| Ser | 1.84 | 4.35 | 1.497 | 3.7 | 0.793 | 3.16 | 0.891 | 2.74 | 0.97 | 3.77 | 0.618 | 3.68 |
| Gln | 18.011 | 42.63 | 8.643 | 21.37 | 4.742 | 18.9 | 11.042 | 34.02 | 4.846 | 18.82 | 4.819 | 28.67 |
| Gly | 0.478 | 1.13 | 0.903 | 2.23 | 0.67 | 2.67 | 0.296 | 0.91 | 0.593 | 2.3 | 0.286 | 1.7 |
| Cit | -- | -- | -- | -- | -- | -- | 0.062 | 0.19 | 0.402 | 1.56 | 0.264 | 1.57 |
| Ala | 1.352 | 3.2 | 1.789 | 4.42 | 1.415 | 5.64 | 1.355 | 4.18 | 1.225 | 4.76 | 0.739 | 4.4 |
| Tyr | 0.583 | 1.38 | 1.315 | 3.25 | 0.103 | 0.41 | 0.968 | 2.98 | 0.717 | 2.79 | 0.458 | 2.72 |
| Cys | -- | -- | 0.049 | 0.12 | 0.044 | 0.18 | 0.075 | 0.23 | 0.185 | 0.72 | 0.106 | 0.63 |
| Nva | 0.499 | 1.18 | 0.375 | 0.93 | 0.37 | 1.48 | 0.391 | 1.2 | 0.264 | 1.03 | 0.198 | 1.18 |
| Hyp | 0.232 | 0.55 | 0.853 | 2.11 | 1.256 | 5 | 0.577 | 1.78 | 0.457 | 1.77 | 0.32 | 1.9 |
| Sar | 0.064 | 0.15 | 0.16 | 0.4 | 0.291 | 1.16 | 0.102 | 0.31 | 0.087 | 0.34 | 0.071 | 0.42 |
| Pro | 1.302 | 3.08 | 6.114 | 15.12 | 2.836 | 11.3 | 3.515 | 10.83 | 4.035 | 15.68 | 2.602 | 15.49 |
| Essential amino acids | 4.555 | 10.77 | 6.637 | 16.4 | 4.789 | 19.08 | 4.665 | 14.37 | 3.896 | 15.15 | 1.754 | 10.43 |
| His | 0.766 | 1.81 | 0.878 | 2.17 | 0.601 | 2.39 | 0.636 | 1.96 | 0.457 | 1.78 | 0.345 | 2.05 |
| Val | 1.695 | 4.01 | 1.934 | 4.78 | 1.365 | 5.44 | 1.409 | 4.34 | 1.152 | 4.48 | 0.078 | 0.46 |
| Met | 0.196 | 0.46 | 0.309 | 0.76 | 0.238 | 0.95 | 0.121 | 0.37 | 0.103 | 0.4 | -- | -- |
| Trp | 0.413 | 0.98 | 0.571 | 1.41 | 0.406 | 1.62 | 0.463 | 1.43 | 0.371 | 1.44 | 0.269 | 1.6 |
| Phe | 0.896 | 2.12 | 1.586 | 3.92 | 1.182 | 4.71 | 1.061 | 3.27 | 0.944 | 3.67 | 0.573 | 3.41 |
| Ile | 0.589 | 1.39 | 1.359 | 3.36 | 0.997 | 3.97 | 0.975 | 3 | 0.869 | 3.38 | 0.489 | 2.91 |
| Semi essential amino acids | 7.099 | 16.8 | 6.801 | 16.81 | 4.219 | 16.81 | 4.671 | 14.39 | 4.662 | 18.11 | 2.392 | 14.23 |
| Thr | 1.324 | 3.13 | 1.478 | 3.65 | 1.085 | 4.32 | 1.029 | 3.17 | 1.02 | 3.96 | 0.604 | 3.59 |
| Arg | 4.195 | 9.93 | 2.21 | 5.46 | 0.188 | 0.75 | 1.957 | 6.03 | 1.59 | 6.18 | 0.968 | 5.76 |
| Leu | 0.802 | 1.9 | 1.872 | 4.63 | 1.492 | 5.95 | 1.049 | 3.23 | 1.273 | 4.95 | 0.638 | 3.8 |
| Lys | 0.778 | 1.84 | 1.241 | 3.07 | 1.454 | 5.79 | 0.636 | 1.96 | 0.779 | 3.02 | 0.182 | 1.08 |
| Total free amino acids | 53.908 |  | 53.883 |  | 34.104 |  | 41.788 |  | 34.3 |  | 20.955 |  |

**Table S3 TAV of free amino acids of different samples**

| Flavor characteristics | Amino acid | Taste threshold/（mg·g^-1^） | TAV | | | | | | Sum | Average value |
| --- | --- | --- | --- | --- | --- | --- | --- | --- | --- | --- |
|  |  |  | 0% | 12% | 14% | 16% | 18% | 20% |  |  |
| Aromatic | Tyr^a^ | 2.6 | 0.22 | 0.51 | 0.04 | 0.37 | 0.28 | 0.18 |  |  |
|  | Phe^ab^ | 0.9 | 1.00 | 1.76 | 1.31 | 1.18 | 1.05 | 0.64 |  |  |
|  | Sum |  | 1.22 | 2.27 | 1.35 | 1.55 | 1.32 | 0.81 | 8.53 | 1.42 |
| Bitter | Arg | 0.5 | 8.39 | 4.42 | 0.38 | 3.91 | 3.18 | 1.94 |  |  |
|  | Val^b^ | 0.4 | 4.24 | 4.83 | 3.41 | 3.52 | 2.88 | 0.19 |  |  |
|  | Trp^bc^ | 0.9 | 0.46 | 0.63 | 0.45 | 0.51 | 0.41 | 0.30 |  |  |
|  | Ile^b^ | 0.9 | 0.65 | 1.51 | 1.11 | 1.08 | 0.97 | 0.54 |  |  |
|  | Leu^b^ | 1.9 | 0.42 | 0.99 | 0.79 | 0.55 | 0.67 | 0.34 |  |  |
|  | Met^c^ | 0.3 | 0.65 | 1.03 | 0.79 | 0.40 | 0.34 | 0.00 |  |  |
|  | Sum |  | 14.82 | 13.41 | 6.93 | 9.99 | 8.45 | 3.31 | 56.90 | 9.48 |
| Sweet | Ser | 1.5 | 1.23 | 2.49 | 0.53 | 0.59 | 0.65 | 0.41 |  |  |
|  | His | 0.2 | 3.83 | 4.39 | 3.00 | 3.18 | 2.29 | 1.72 |  |  |
|  | Thr^b^ | 2.6 | 0.51 | 0.57 | 0.42 | 0.40 | 0.39 | 0.23 |  |  |
|  | Pro | 3.0 | 0.43 | 2.04 | 0.95 | 1.17 | 1.35 | 0.87 |  |  |
|  | Sum |  | 6.00 | 9.49 | 4.89 | 5.34 | 4.67 | 3.24 | 33.63 | 5.61 |
| Umami | Asp^a^ | 1.0 | 2.81 | 1.44 | 1.09 | 0.87 | 0.96 | 0.50 |  |  |
|  | Glu^a^ | 0.3 | 1.94 | 6.26 | 4.36 | 3.29 | 4.05 | 2.49 |  |  |
|  | Gly^a^ | 1.3 | 0.37 | 0.69 | 0.52 | 0.23 | 0.46 | 0.22 |  |  |
|  | Ala^a^ | 0.6 | 2.25 | 2.98 | 2.36 | 2.26 | 2.04 | 1.23 |  |  |
|  | Lys^bc^ | 0.5 | 1.56 | 2.48 | 2.91 | 1.27 | 1.56 | 0.36 |  |  |
|  | Sum |  | 8.92 | 13.86 | 11.24 | 7.92 | 9.06 | 4.80 | 55.81 | 9.30 |

Different letters (a, b, c, e and f) in the same line indicate significant differences (P＜0.05).

**Table S4 Threshold, ROAV and odor description of volatile components of fresh and fermented rape stalks with different salt concentrations**

| Name | Threshold　(μg/kg) | The fresh samples | | 12% | | 14% | | 16% | | 18% | | 20% | | Odor description |
| --- | --- | --- | --- | --- | --- | --- | --- | --- | --- | --- | --- | --- | --- | --- |
|  |  | Relative content/% | ROAV | Relative content /% | ROAV | Relative content /% | ROAV | Relative content /% | ROAV | Relative content /% | ROAV | Relative content /% | ROAV |  |
| 2-isothiocyanate ethyl benzene | 100 | 0.82 | 0.84 | -- | -- | -- | -- | -- | -- | 0.37 | <0.1 | -- | -- |  |
| Benzyl cyanide | 100 | -- | -- | -- | -- | -- | -- | 0.15 | <0.1 | -- | -- | 0.11 | <0.1 |  |
| 1-octen-3-ol | 1 | 0.43 | 44.48 | 0.25 | 12.44 | -- | -- | 0.21 | 0.19 | 0.23 | 0.39 | 0.25 | 3.18 | Mushroom flavor, green flavor and vegetable flavor |
| Benzylalcohol | 10000 | -- | -- | 1.98 | <0.1 | 1.14 | <0.1 | 1.67 | <0.1 | 1.67 | <0.1 | 1.39 | <0.1 | Aromatic flavor |
| Phenylethanol | 10000 | -- | -- | 9.56 | <0.1 | 4.31 | <0.1 | 9.28 | <0.1 | 6.04 | <0.1 | 5.95 | <0.1 | Hyacinth and gardenia aroma |
| Heptanol | 200 | 0.88 | 0.45 | -- | -- | 0.34 | <0.1 | 0.57 | <0.1 | 0.46 | <0.1 | 0.60 | <0.1 | Fruity, mellow |
| Pentanol | 4000 | 1.21 | <0.1 | 0.13 | <0.1 | 0.12 | <0.1 | -- | -- | -- | -- | -- | -- | Aroma of bread, wine and fruit |
| N-hexanol | 2500 | 0.30 | <0.1 | 0.09 | <0.1 | 0.12 | <0.1 | -- | -- | 0.11 | <0.1 | 0.48 | <0.1 | Green, fruity, mellow, sweet, ethereal |
| 2,6-dimethoxyphenol | 1850 | 0.14 | <0.1 | -- | -- | -- | -- | -- | -- | -- | -- | -- | -- | Pungent fragrance |
| phenol | 5900 | -- | -- | 0.60 | <0.1 | 2.49 | <0.1 | 4.55 | <0.1 | 1.08 | <0.1 | 0.14 | <0.1 | Phenol aroma, medicine aroma |
| 4-methylthionitrile | 50 | 0.33 | 0.68 | -- | -- | -- | -- | -- | -- | -- | -- | -- | -- |  |
| 5-hexanitrile | 10000 | 1.39 | <0.1 | -- | -- | -- | -- | 1.43 | <0.1 | -- | -- | 0.38 | <0.1 | unsavory |
| Benzopropiononitrile | 500 | 11.30 | 2.32 | 1.43 | 0.14 | 1.61 | <0.1 | 30.90 | <0.1 | 2.50 | <0.1 | 23.83 | 0.60 | Unsavory |
| Dimethyl trisulfide | 0.01 | -- | -- | -- | -- | 1.27 | 100.00 | 1.06 | 100.00 | 0.59 | 100.00 | 0.08 | 100.00 | Areca, sulfur |
| naphthalene | 60 | 0.59 | 1.01 | 0.20 | 0.16 | 0.43 | <0.1 | 0.58 | <0.1 | 0.64 | <0.1 | 0.90 | 0.19 | tarry |
| furan | 6 | 0.35 | 6.03 | 0.38 | 3.17 | 0.89 | 0.12 | 1.56 | 0.24 | 0.85 | 0.24 | 0.53 | 1.09 |  |
| Pyrazine | 1000 | -- | -- | -- | -- | 0.14 | <0.1 | -- | -- | -- | -- | -- | -- |  |
| (E,E) - 2,4-heptadiene aldehyde | 30 | 0.92 | 3.16 | 0.41 | 0.68 | 0.33 | <0.1 | 0.17 | <0.1 | 0.44 | <0.1 | 0.54 | 0.23 | Nut fat flavor |
| 2-pentenoaldehyde | 1500 | 0.16 | <0.1 | -- | -- | -- | -- | -- | -- | -- | -- | -- | -- | Waxy, green and fruity |
| Benzaldehyde | 3500 | 3.61 | 0.11 | 2.95 | <0.1 | 3.45 | <0.1 | 3.46 | <0.1 | 3.27 | <0.1 | 3.08 | <0.1 | Greasy sweetness, almond aroma, fruit aroma |
| Phenylacetaldehyde | 4 | 2.37 | 60.85 | 5.43 | 67.17 | 5.70 | 1.12 | 5.56 | 1.31 | 3.83 | 1.62 | 2.03 | 6.34 | Green, rose, flower, chocolate |
| Decanal | 2 | 0.68 | 34.72 | -- | -- | 0.12 | <0.1 | 0.23 | 0.11 | 0.19 | 0.16 | 0.22 | 1.36 | Aldehyde, wax, fat and citrus aroma |
| Hexanal | 4500 | 1.11 | <0.1 | 0.09 | <0.1 | 0.16 | <0.1 | 0.12 | <0.1 | 0.17 | <0.1 | 0.19 | <0.1 | Green fragrance, leaf fragrance, fruit fragrance and wood fragrance |
| furfural | 3 000 | 0.15 | <0.1 | 0.45 | <0.1 | 0.12 | <0.1 | 0.15 | <0.1 | 0.12 | <0.1 | 0.12 | <0.1 | Bitter almond and caramel |
| Nonanal | 4 | 2.56 | 65.74 | -- | -- | -- | -- | 0.65 | 0.15 | -- | -- | 0.84 | 2.62 | Oil and grass flavor |
| Pentadecanal | 1.48 | 0.30 | 20.78 | 0.07 | 2.48 | -- | -- | 0.31 | 0.20 | -- | -- | 0.14 | 1.21 |  |
| Phenylacetic acid | 10 000 | -- | -- | -- | -- | 0.09 | <0.1 | -- | -- | 00.13 | <0.1 | -- | -- | Special smell |
| butyrate | 240 | -- | -- | 0.33 | <0.1 | 0.30 | <0.1 | 0.43 | <0.1 | -- | -- | -- | -- |  |
| Caproic acid | 3 000 | -- | -- | -- | -- | 0.47 | <0.1 | -- | -- | -- | -- | 0.54 | <0.1 | Sweat smell |
| Nonanoic acid | 1000 | -- | -- | 0.23 | <0.1 | -- | -- | 0.42 | <0.1 | -- | -- | -- | -- |  |
| Hexadecanoic acid | 10000 | -- | -- | 0.29 | <0.1 | 0.24 | <0.1 | 0.28 | <0.1 | 0.14 | <0.1 | -- | -- |  |
| Octoic acid | 5 000 | -- | -- | 1.01 | <0.1 | 0.91 | <0.1 | 2.59 | <0.1 | 1.63 | <0.1 | 1.71 | -- | Lanolin, boiled peanut flavor |
| N-valeric acid | 3000 | -- | -- | 0.49 | <0.1 | 0.15 | <0.1 | -- | -- | 0.37 | <0.1 | 0.10 | <0.1 | Sweet, cheese |
| palmitic acid | 10 000 | 0.13 | <0.1 | 0.85 | <0.1 | 0.74 | <0.1 | 0.97 | <0.1 | 0.48 | <0.1 | 0.36 | <0.1 |  |
| 2-butanone | 50000 | -- | -- | 0.15 | <0.1 | -- | -- | -- | -- | -- | -- | 0.07 | <0.1 | Ethereal and fruity |
| 2-heptanone | 140 | -- | -- | -- | -- | 0.14 | <0.1 | 0.09 | <0.1 | 0.78 | <0.1 | -- | -- | Banana, cheese aroma and slight medicine aroma |
| 2-decanone | 9 | -- | -- | 2.11 | 11.61 | 2.20 | 0.19 | 1.37 | 0.14 | 2.12 | 0.40 | 1.32 | 1.83 |  |
| β- Ionone | 10 | 9.75 | 100.00 | 9.92 | 49.13 | 10.99 | 0.87 | 8.04 | 0.76 | 14.18 | 2.40 | 14.65 | 18.31 |  |
| Furanone | 40 | 1.60 | 4.12 | 5.30 | 6.56 | 6.29 | 0.12 | 2.26 | <0.1 | 5.79 | 0.25 | 4.30 | 1.34 | Sweet fruit, caramel flavor |
| Heptanone | 50 | 0.38 | 0.78 | 0.50 | 0.50 | 0.53 | <0.1 | -- | -- | -- | -- | 0.22 | <0.1 |  |
| Hexanone | 50000 | -- | -- | 0.65 | <0.1 | 0.45 | <0.1 | 0.24 | <0.1 | 0.47 | <0.1 | -- | -- | Sweet, fruity and waxy |
| Dodecane | 2 040 | 8.45 | <0.1 | 4.60 | <0.1 | 3.20 | <0.1 | 7.87 | <0.1 | 4.53 | <0.1 | 5.62 | <0.1 | Greasy and fishy smell |
| Tridecane | 2 140 | 1.68 | <0.1 | 1.48 | <0.1 | 0.46 | <0.1 | 2.46 | <0.1 | 0.68 | <0.1 | 1.25 | <0.1 |  |
| Tetradecane | 1 000 | 0.74 | <0.1 | 0.61 | <0.1 | -- | -- | 1.69 | <0.1 | 0.77 | <0.1 | 0.38 | <0.1 |  |
| N-Heptadecane | 300 000 | -- | -- | 0.16 | <0.1 | 0.15 | <0.1 | 0.34 | <0.1 | -- | -- | 0.73 | <0.1 |  |
| D-limonene | 34 | -- | -- | -- | -- | -- | -- | 0.10 | <0.1 | -- | -- | -- | -- |  |
| glycerol | 4400000 | -- | -- | -- | -- | -- | -- | -- | -- | 0.19 | <0.1 | 0.14 | <0.1 |  |
| Ethyl decanoate | 0.2 | -- | -- | -- | -- | 0.29 | 1.14 | 0.33 | 1.53 | -- | -- | 0.33 | 20.89 |  |
| Ethyl laurate | 3.5 | -- | -- | -- | -- | 0.38 | <0.1 | 0.27 | <0.1 | 0.11 | <0.1 | 0.24 | 0.86 | Fruity and floral |
| Methyl palmitate | 4 | 0.29 | 7.39 | 1.35 | 16.68 | 1.25 | 0.25 | 0.69 | 0.16 | 1.02 | 0.43 | 0.52 | 1.63 | Ester flavor and mellow flavor |
| Ethyl palmitate | 1.5 | -- | -- | 3.03 | 100.00 | 3.75 | 1.97 | 1.63 | 1.03 | 1.69 | 1.91 | 1.14 | 9.54 | Cream flavor, ester flavor |
