# Supplementary figures and images for: Properties investigations of rape stalks fermented by different salt concentration: Effect of volatile compounds and physicochemical indexes
Source: Food Chem X. 2023 Jun 10;18:100746. doi: 10.1016/j.fochx.2023.100746 (PMC10314211; doi:10.1016/j.fochx.2023.100746)

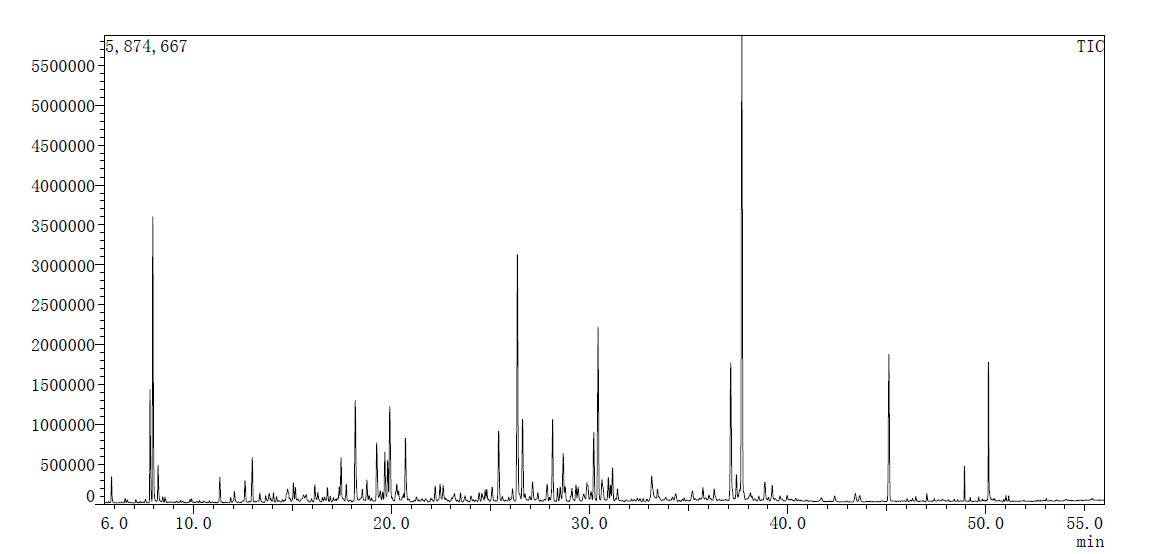

Supplement: Supplementary data 2 [file mmc2.zip › GCMS fresh sample chromatogram.png]

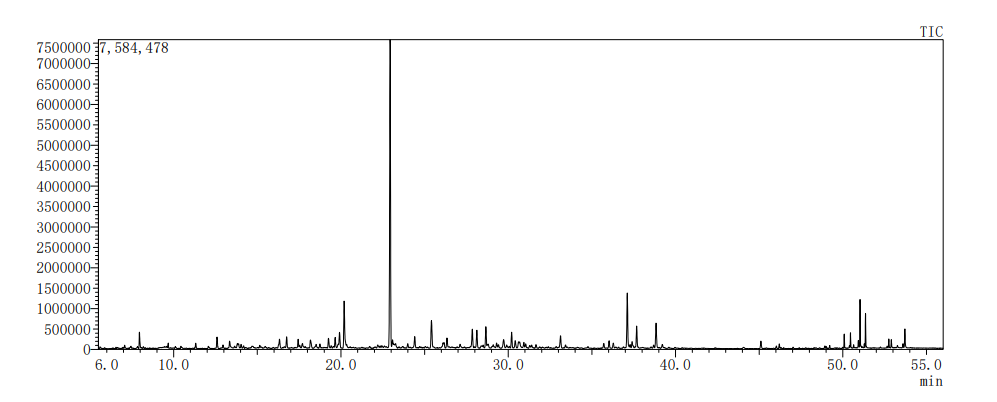

Supplement: Supplementary data 2 [file mmc2.zip › GCMS12% sample chromatogram.png]

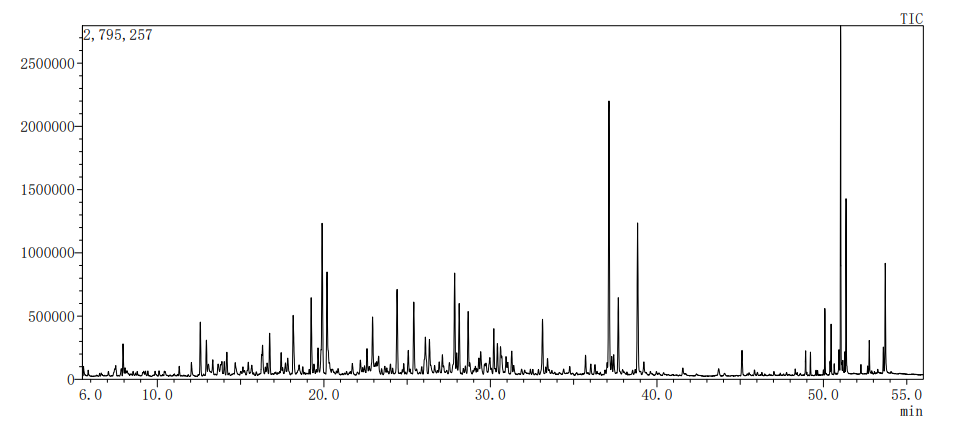

Supplement: Supplementary data 2 [file mmc2.zip › GCMS14% sample chromatogram.png]

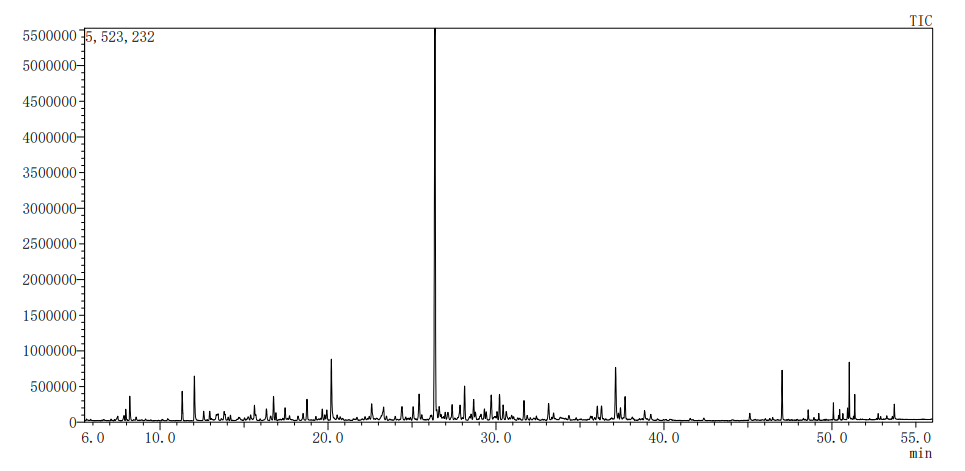

Supplement: Supplementary data 2 [file mmc2.zip › GCMS16% sample chromatogram.png]

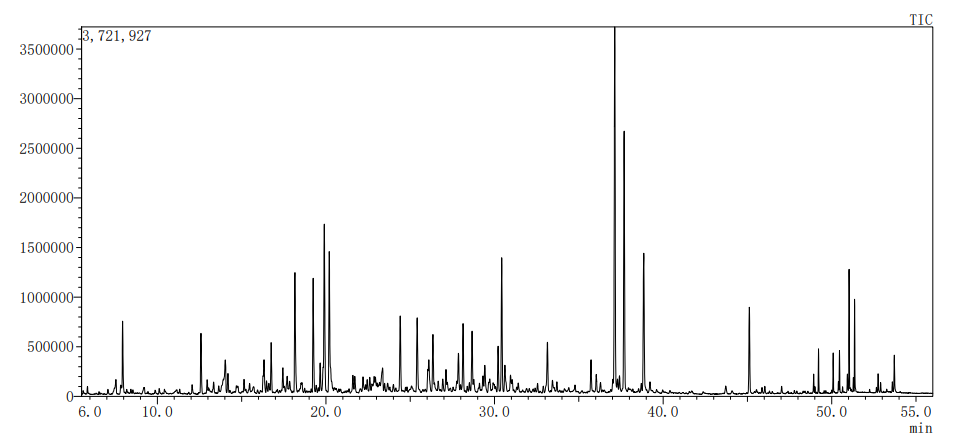

Supplement: Supplementary data 2 [file mmc2.zip › GCMS18% sample chromatogram.png]

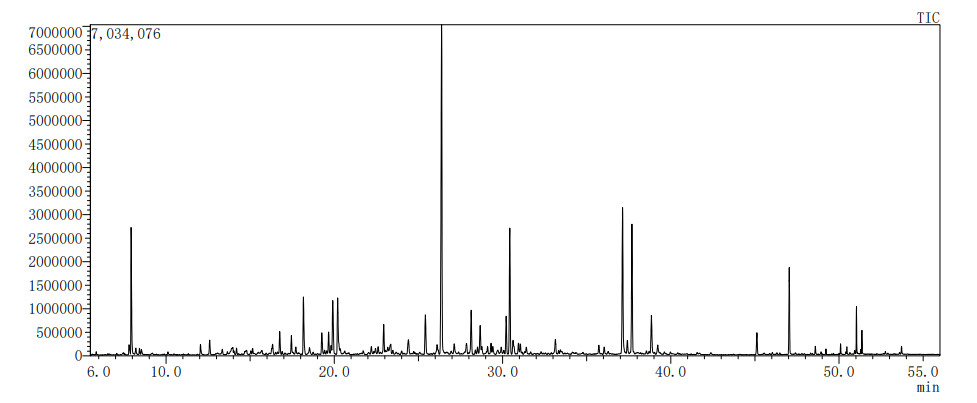

Supplement: Supplementary data 2 [file mmc2.zip › GCMS20% sample chromatogram.png]

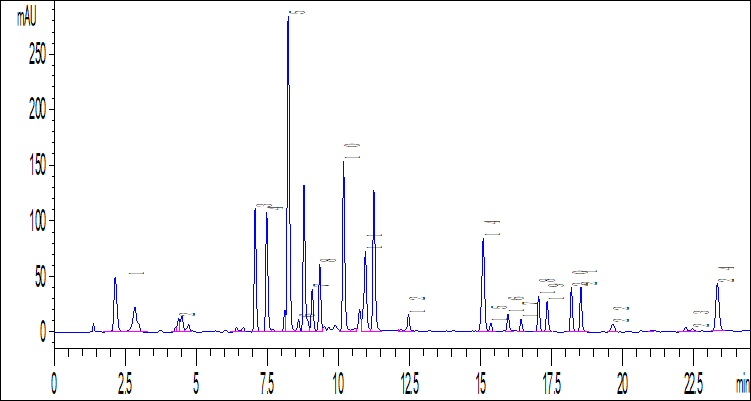

Supplement: Supplementary data 2 [file mmc2.zip › HPLC fresh sample chromatogram.png]

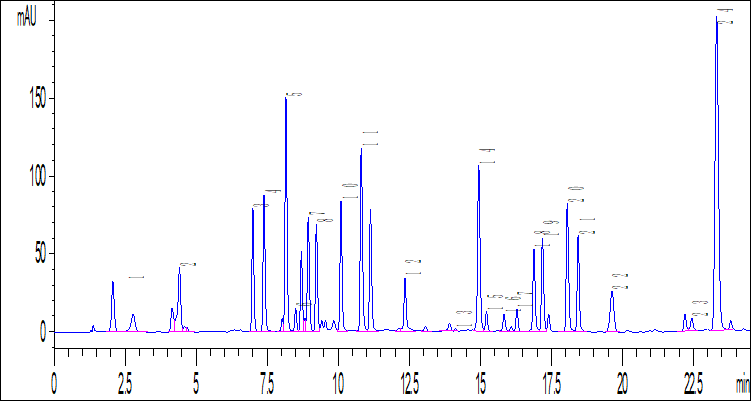

Supplement: Supplementary data 2 [file mmc2.zip › HPLC12% sample chromatogram.png]

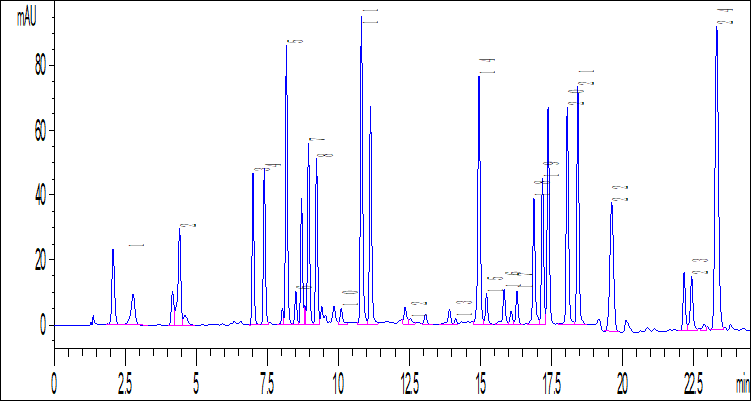

Supplement: Supplementary data 2 [file mmc2.zip › HPLC14% sample chromatogram.png]

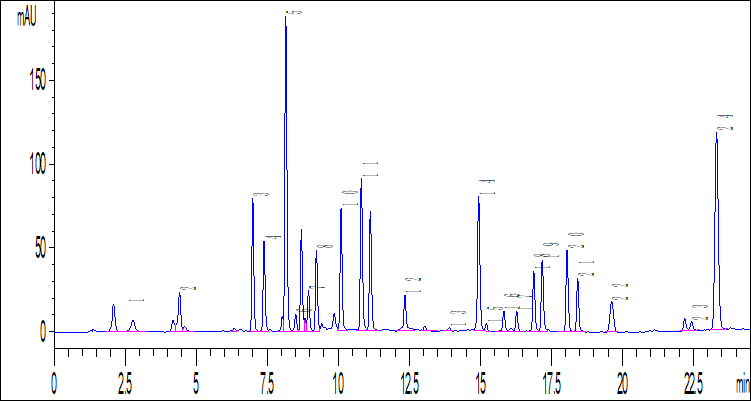

Supplement: Supplementary data 2 [file mmc2.zip › HPLC16% sample chromatogram.png]

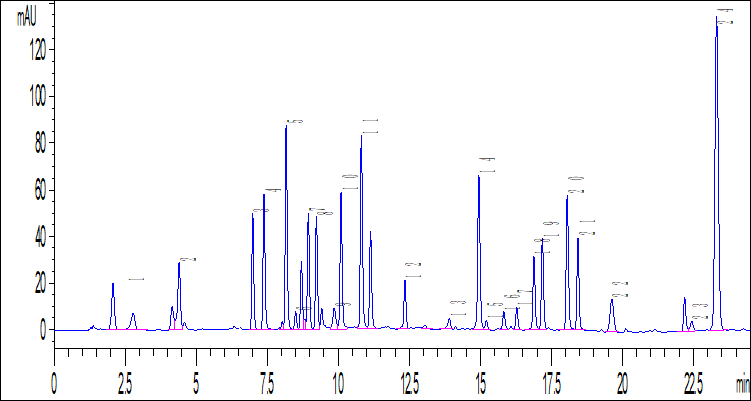

Supplement: Supplementary data 2 [file mmc2.zip › HPLC18% sample chromatogram.png]

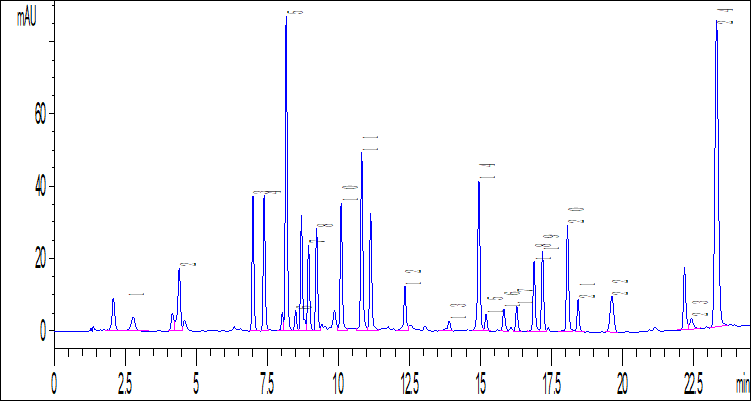

Supplement: Supplementary data 2 [file mmc2.zip › HPLC20% sample chromatogram.png]
